# Allogeneic Embryos Disregulate Leukemia Inhibitory Factor (LIF) and Its Receptor in the Porcine Endometrium During Implantation

**DOI:** 10.3389/fvets.2020.611598

**Published:** 2020-11-24

**Authors:** Josep M. Cambra, Amaia Jauregi-Miguel, Manuel Alvarez-Rodriguez, Inmaculada Parrilla, Maria A. Gil, Emilio A. Martinez, Cristina Cuello, Heriberto Rodriguez-Martinez, Cristina A. Martinez

**Affiliations:** ^1^Department of Medicine and Animal Surgery, Faculty of Veterinary Medicine, International Excellence Campus for Higher Education and Research “Campus Mare Nostrum”, University of Murcia, Murcia, Spain; ^2^Institute for Biomedical Research of Murcia (IMIB-Arrixaca), Campus de Ciencias de la Salud, Murcia, Spain; ^3^Department of Biomedical & Clinical Sciences (BKV), BKH/Obstetrics & Gynaecology, Faculty of Medicine and Health Sciences, Linköping University, Linköping, Sweden; ^4^Wallenberg Centre for Molecular Medicine, Linköping University, Linköping, Sweden

**Keywords:** LIF, LIF receptor, embryo transfer, endometrium, immunotolerance, allogeneic pregnancy, pig

## Abstract

Despite its advantages for pig breeding, embryo transfer (ET) has a major handicap: high embryo mortality during the pre- and implantation period, probably caused by divergent phenomena of tolerance between the immunologically unrelated (i.e., allogeneic) embryos and the recipient sow. Thus, to reach a similar maternal tolerance as in conventional breeding by artificial insemination (AI) would be the key to ET-success. For this reason, we studied the expression of the leukemia inhibitory factor (LIF) cytokine and its receptor in the pig endometrium during the implantation period (days 18 and 24) in sows subjected to ET (AL group) vs. post-cervical-AI controls (Hemi-AL group). Quantification of expression was performed at both mRNA (rt-qPCR) and protein (WB) levels. The expression of endometrial LIF on day 24 was considerably lower in ET than in AI pregnancies. Correlations between endometrial mRNA levels of LIF and LIF-R showed that, contrary to early AI-pregnancies, ET-pregnancies lack an inverse relation between cytokine and receptor levels. In conclusion, ET-pregnancies lack sufficient endometrial levels of LIF to develop adequate immunotolerance mechanisms to prevent the rejection of allogeneic ET-embryos.

## Introduction

The implementation of emergent breeding technologies in pig production, as embryo transfer (ET), would support sustainability and competitiveness of the commercial pig sector. ET-technology has demonstrated considerable advantages for the introduction of new genetic material into high health status herds, with a minimal risk of disease transmission, without affecting animal welfare during transport and reduction of transportation costs (1). In addition, ET is a pre-requisite for developing other biotechnologies, as cloning or gene editing (2), with the pig being one of the most accepted large animal models in biomedical research because of its anatomical and genomic resemblance to human (3).

Recent advances in porcine ET technology have allowed its application in the porcine industry today (1). However, the reproductive performance of recipients after ET continue to be suboptimal, mostly due to the high embryo mortality depicted (>50%), compared to natural breeding or artificial insemination (AI) (4, 5), particularly during the implantation period (Days 15–30). During this period, embryos are responsible of triggering the so-called maternal immune tolerance to the embryo and placental allografts (6). After natural mating or AI, the maternal environment is exposed to hemi-allogeneic embryos, i.e., embryos containing allo-antigens from the paternal side only. In the case of ET, fully allogeneic embryos (with both maternal and paternal allo-antigens, immunologically differing from the recipient female) are transferred into the genital tract, probably affecting the mechanisms regulating maternal immune tolerance and leading to affected embryo survival rates. Of interest, this process is also relevant for humans considering the increasing use of surrogate recipients, involving ET into a recipient who is immunologically distant from the biological parents, i.e., receiving an allogeneic embryo. In this respect, a study in pigs might be of interest, particularly during the process of implantation, being, in contrast to human, central and non-invasive in porcine (7), suggesting that an adequate balance between pro- and anti-inflammatory cytokines during the implantation period is essential in pigs to reach a satisfactory immune response to the allografts (8). The Leukemia inhibitory factor (LIF), part of the Interleukine-6 (IL-6) family of cytokines (9) appears to be relevant during this period due to its role as an uterine immune modulator, as proven in mice (10).

During the implantation window, endometrial LIF expression increases considerably, as has been reported in different mammals such as humans (9, 11), mice (12) and pigs (13). Although the biology of endometrial LIF-actions is not fully understood, reports suggest this cytokine is involved in several events during implantation such as regulation of uterine leukocyte infiltration (14), embryo-endometrial interaction (9) and uterine receptivity (15), among others. In addition, LIF seems to be a good predictor of fertility after *in vitro* fertilization (IVF) treatments, since high levels of LIF endometrial expression during the mid-luteal phase relates to a higher chance of pregnancy success in women that underwent IVF (16).

The IL-6 family of cytokines (including LIF) shares a specific receptor called LIF-R (17), with recognized roles in reproductive processes (18). LIF and LIF-R expression have been previously reported in the porcine endometrium and placenta (13, 19–21). These authors described their expression patterns during the estrous cycle and pregnancies performed by AI but, to the best of our knowledge, there are not previous studies about LIF or LIF-R expression in pregnancies stablished after ET. Therefore, the aim of this study was to compare endometrial gene and protein expression of LIF and LIF-R between pregnancies obtained by AI (hemi-allogeneic embryos) and ET employing donor embryos (allogeneic embryos) throughout the implantation period.

## Materials and Methods

Unless otherwise specified, reagents were obtained from Sigma-Aldrich Quimica SA (Madrid, Spain). All animal procedures complied with the European Directive 2010/63/EU for animal experiments and were a priori examined and approved by the Ethical Committee for Experimentation with Animals of Murcia University (research code: 522/2019).

### Animals

Animals selected for this experiment were multiparous (2–7 parity) crossbred sows from the same genetic line (Landrace x Large-White) with similar lactation periods (21–24 d). After weaning, the females were individually housed to crates in a mechanically ventilated facility (Agropor SA, Murcia, Spain). Semen for the AIs (Hemi-AL and embryo donors) was obtained from sexually mature boars (2–3 years of age) of proven fertility. All animals had *ad libitum* access to water and were fed twice a day with commercial feedstuff.

### Estrus Detection and Artificial Insemination

Estrus detection started the same day after weaning and was performed by skilled operators applying backpressure during exposing the females to snout-to-snout contact with a mature boar. Sows that exhibited a clear standing reflex were considered in heat and the day of the onset of estrus was defined as Day 0 (D0). Only sows that started the estrus at Day 4 (D4) or Day 5 (D5) after weaning were selected for the experiments. Females were post-cervically inseminated at 12 and 24 h after the onset of estrus with refrigerated doses containing 1.5 × 10^9^ spermatozoa, extended in 40 mL of Beltsville thawing solution (22).

### Collection, Evaluation, and Transfer of Embryos

For embryo collection, donor sows were subjected to laparotomies at D5 in a surgical room located on the farm. Donors were sedated by administration of azaperone (2 mg/kg body weight, intramuscular) and subsequently, general anesthesia was induced with sodium thiopental (7 mg/kg body weight, intravenous) and maintained with isoflurane (3–5%). After exposure of the genital tract and once the number of corpora lutea on the ovaries was counted, the tip of each uterine horn was flushed with 30 mL of modified Tyrode's lactate (TL)-HEPES-polyvinyl alcohol (PVA) (TL-HEPES-PVA) (23) medium at 37°C. For the flushing, a blunt needle connected to a syringe filled with flushing medium was inserted through the uterus near to the utero-tubal junction. At the other extreme (about 35 cm from the first incision) the wall of the uterus was perforated with an Adson forceps and a curved glass catheter was inserted. The walls of the uterus were manually compressed, helping the medium to flow through the uterine tract into the collection catheter and recovered in a collection tube. After embryo collection, the developmental stage and quality of embryos were assessed according to the criteria determined by the International Embryo Transfer Society (24). Only compacted morulae, morphologically classified as good or excellent, were used in the experiment. After embryo collection and assessment, embryos were surgically transferred into recipients at D5 of the cycle with a procedure similar to that described for embryo collection. Before ET, each recipient received a single injection of a long-acting amoxicillin suspension (Clamoxyl LA; Pfizer, Madrid, Spain; 15 mg/ kg, intramuscular). A total of 50 μL of TL-HEPES-PVA containing pools of 23 morulae were loaded into Tomcat catheters separated by air bubbles from another 30 μL of medium on the front and back. The loaded catheters were inserted through the uterine wall at the tip of one of the uterine horns (~15 cm below the utero-tubal junction) and the contents introduced into the uterus with the help of a 1 mL syringe attached to the catheter. The number of embryos transferred per sow was based on the mean number of corpora lutea accounted in the Hemi-AL group (23.5 + 1.0), thus ensuring that the number of embryos was similar in both experimental groups.

### Tissue Collection

The uterine wall was opened along the anti-mesometrial side to avoid disrupting conceptus attachment sites. Three strips of tissue were collected from each ad-mesometrial region: site of implantation (IMP) and site of non-implantation (Non-IMP; only D24). The strips were obtained by cutting the uterine tissue of the mentioned regions with a surgical blade consisting only of endometrial tissue, avoiding the muscular or serous layers. The size was estimated to be 2–4 mm wide and the weight ~20 mg. Once taken, each strip was washed 3 times in PBS to remove blood traces and dried in a sterile paper to remove excess medium. Tissue samples were placed in microtubes containing RNA later and stored at −80°C until further analysis.

### RNA Isolation and Reverse Transcription

Total RNA from endometrial tissues was extracted with Trizol reagent according to manufacturer's instructions (Thermo Scientific, Waltham, MA, USA) and checked for quantity using a NanoDrop system (Thermo Scientific NanoDrop2000) and for quality using an Agilent 2100 bioanalyzer (Agilent Technologies, Palo Alto, CA, USA). The RNA integrity number (RIN) of the samples was between 7 and 10. Then, RNA was reverse-transcribed to cDNA using the High-Capacity cDNA Reverse Transcription Kit (Applied Biosystem, CA, USA) at 25°C for 10 min, 37°C for 120 min followed by 85°C for 5 s in a Thermal cycler (BioRad-DNA Engine, Hercules, CA, USA).

### Real Time Quantitative Polymerase Chain Reaction (RT-qPCR)

Relative mRNA expression of LIF and LIF-R in endometrial samples were quantified by q-PCR using the Real-Time PCR Detection System (CFX9; Bio-Rad Laboratories, Inc.; CA, USA). Primers for LIF, LIF-R and GAPDH (housekeeping) genes were designed with the software primer3plus (25). Primers were designed to cross exon-exon boundaries to ensure that the amplified product is not generated from genomic DNA contamination. For increased sensitivity, *GAPDH* primers were specially designed to not differentiate between the isoforms. The specificity of the primers was checked using a BLAST analysis against the genomic NCBI database. Primer pairs details are listed in Table 1. PowerUp SYBR Green Master Mix (2X) (Applied Biosystems, CA, USA) was used for PCR reactions. The final reaction volume of 10 μL included 2 μL of cDNA (25 ng), 5 μL of 2X Master mix, 1 μL of each primer (500 nM), and 1 μL of dH_2_O. The following PCR conditions were used: initial UDG activation of 50°C for 2 min and a previous denaturation of 95°C for 2 min. Those steps were followed by 40 cycles of 5 s of denaturation at 95°C, and 30 s of extension and annealing at 60°C. qPCRs were run in duplicate for each gene per sample. The specificity of the q-PCR was confirmed by detection of a single distinct peak on examination of the dissociation curve profile of the reaction product and the analysis of amplicon size by agarose gel electrophoresis. The relative gene expression was calculated using the ΔΔCt method applying the efficiency correction technique described by Pfaffl (26). Target gene expression was normalized with the housekeeping reference gene glyceraldehyde-3-phosphate dehydrogenase (*GAPDH*). Efficiency for each primer pair, was calculated obtaining the slope of the regression line of the Ct values obtained in a 5 serial sample dilution, according to the equation: E = 10^[−1/slope]^.

**Table 1:** Primer sequences for the analysis of mRNA gene expression.

**GENE**	**Forward (5^′^ → 3^′^)**	**Reverse (5^′^ → 3^′^)**	**Size**	**Efficiency**	**References**
LIF	GCCAACGCCCTCTTTATTCT	GTTCACAGCACCAGGATTGA	222	102.1	AY585336.1
LIF-R	GGTCGCAAAGAGTGGAGTGA	TTCTGCCAATCTGTGCCGAT	163	87.0	XM_021076925.1 (21)
GAPDH	ATCACTGCCACCCAGAAGAC	AGATCCACAACCGACACGTT	194	96.6	NM_001206359.1

### Western Blotting (WB)

Protein extraction and Western blotting were performed as previously described (27). Briefly, total protein from endometrial samples were extracted in a commercial lysis buffer (RIPA) containing protease inhibitors (Thermofisher, Rockford, USA), homogenized by sonication (50 W, 60 sec) and then kept at 4°C for 60 min in rotation. After centrifugation (13,000 xg, 4°C for 10 min), supernatants were collected and quantified by the DC Protein Assay Kit (Applied Biosystems). Then, 25 μg of each sample were loaded into 4–20% SDS-PAGE Mini-PROTEAN TGX Precast Protein Gels (BioRad, Richmond, CA, USA) and transferred to polyvinyldifluoride (PVDF) membranes (BioRad). Prior to immunoblotting, membranes were blocked with Odyssey blocking buffer solution (LI-COR Biosciences, Lincoln, NE, USA) at room temperature for 60 min in rotation. After extensive washing in Tris buffering solution (TBS) with 0.1% Tween 20, membranes were incubated overnight at 4°C with primary antibodies against LIF (MBS820385, MyBioSources, California, USA) and LIF-R (and ab232877, Abcam, Cambridge, UK) at a dilution of 1/1,000. The anti-β-actin antibody (ab115777, Abcam) was used as an endogenous control at a dilution 1/100 in the same membranes at room temperature during 60 min in rotation.

Specificity of anti-LIF and anti-LIF-R primary antibodies were confirmed incubating each antibody with its respective specific blocking peptide (LIF blocking peptide: MBS9229656, LIF-R blocking peptide: MBS9229656, MyBioSources) at 1:10 ratio. After primary antibody incubations, membranes were washed in TBS with 0.1% Tween 20 and incubated with the secondary goat anti-mouse IRDye 800 CW (925-32210; LI-COR Biosciences) at room temperature during 60 min. Finally, after membrane scanning using the Odyssey CLx (LI-COR Biosciences), blots images were obtained using the image studio 4.0 software (LI-COR Biosciences).

### Statistical Analyses

The relative expression of mRNA or protein is expressed as the mean ± the standard error of the mean (SEM). The normality of the variables was analyzed by the Kolmogorov-Smirnov or Shapiro-Wilk tests according to the sample number. The statistical significance was determined using Student's *t*-test corrected for inequality of variances with the Levene's test. Correlations between the relative mRNA expression of LIF and LIF-R were compared using the Pearson's correlation. These results are shown as Pearson's correlation and the *p*-value. All statistical analyses were performed using the IBM SPSS 24.0 Statistics package software (SPSS, Chicago, IL, USA). Differences were considered significant at *p* < 0.05.

### Experimental Design

**Figure 1:**
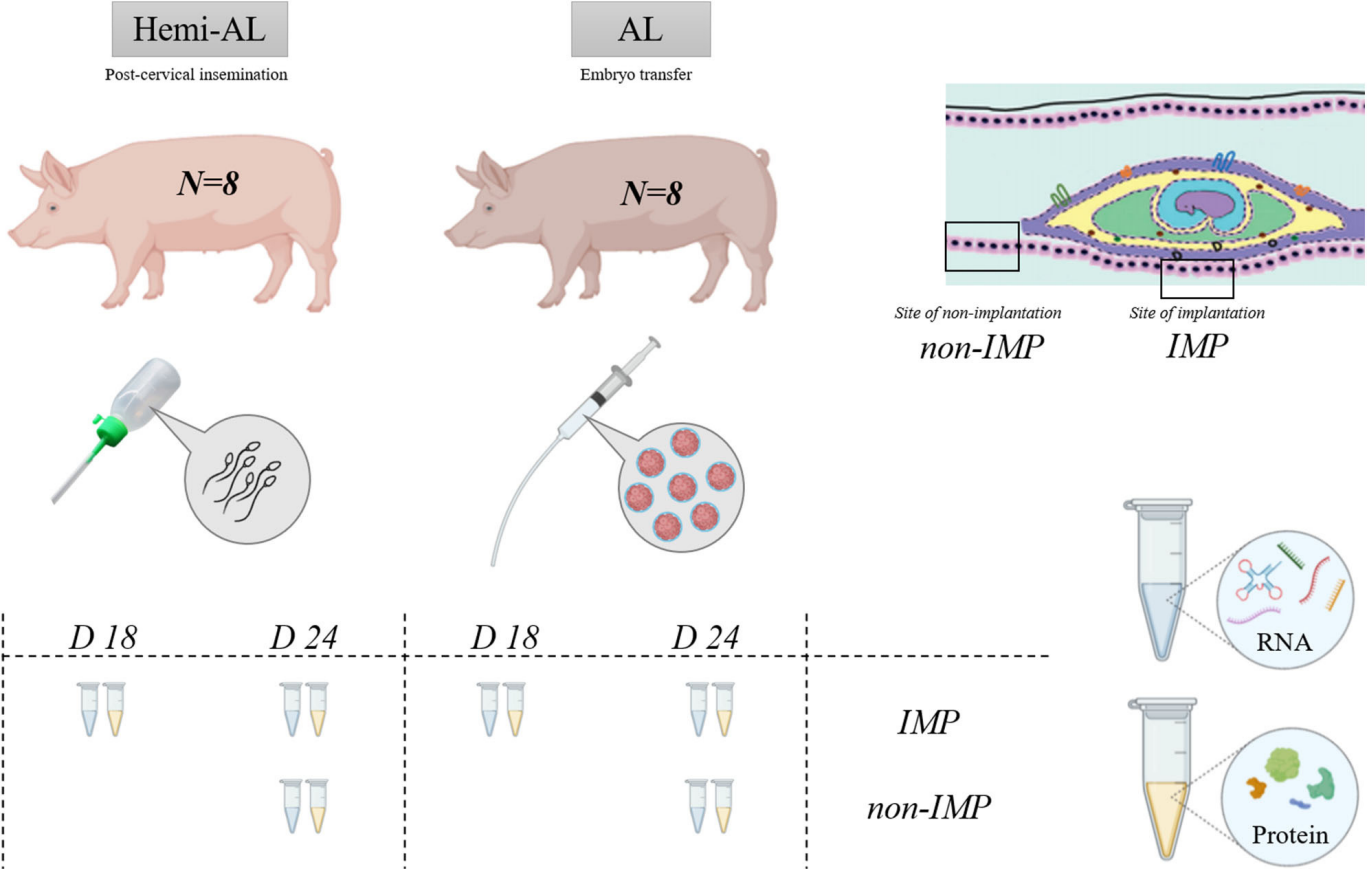
Schematic representation of the experimental design. Two experimental groups were used in the experiment: Hemi-AL (post-cervical artificial insemination), AL (embryo transfer employing donor embryos). Tissues analyzed include endometrial areas of days 18 (D18) and 24 (D24) after the onset of the estrus, collected from implantation areas (IMP) and non-implantation (Non-IMP) areas. Total RNA and protein were extracted for calculating LIF and LIF-R gene and protein expression.

As represented in Figure 1, gene and protein expression levels of LIF and LIF-R were studied in hemi-allogeneic pregnancies obtained through post-cervical AI in sows containing embryos with maternal genetic material; Hemi-AL group; *n* = 8) or allogeneic pregnancies (recipient sows subjected to ET, so containing embryos from parents genetically different from the recipient sows; Donor embryo group, AL; *n* = 8). In order to simulate the experimental conditions of the surgical ET, Hemi-AL sows were also subjected to surgical sham-interventions on the same day of ET. All sows were examined for signs of estrus beginning 12 days post-surgery, until the day of endometrial collections. Samples of ad-mesometrial endometrium were retrieved from pregnant sows on Day 18 (D18) or Day 24 (D24) after the onset of estrus. The tissue samples consisted of pools of three different portions of the endometrium including the epithelial surface and endometrial glands (8). At D24, endometrium was collected from the site of implantation (IMP) and between implantation areas (Non-IMP). Because the embryos were not completely implanted at D18, being randomly distributed throughout the endometrium, samples of non-implantation sites were not collected at this day classifying all samples obtained as areas of implantation. Samples were collected from a total of four animals within each experimental group at both evaluation periods (total *n* = 16).

## Results

### LIF mRNA and Protein Expression Is Altered After Allogeneic Embryo Transfer at the Endometrial Implantation Sites

**Figure 2:**
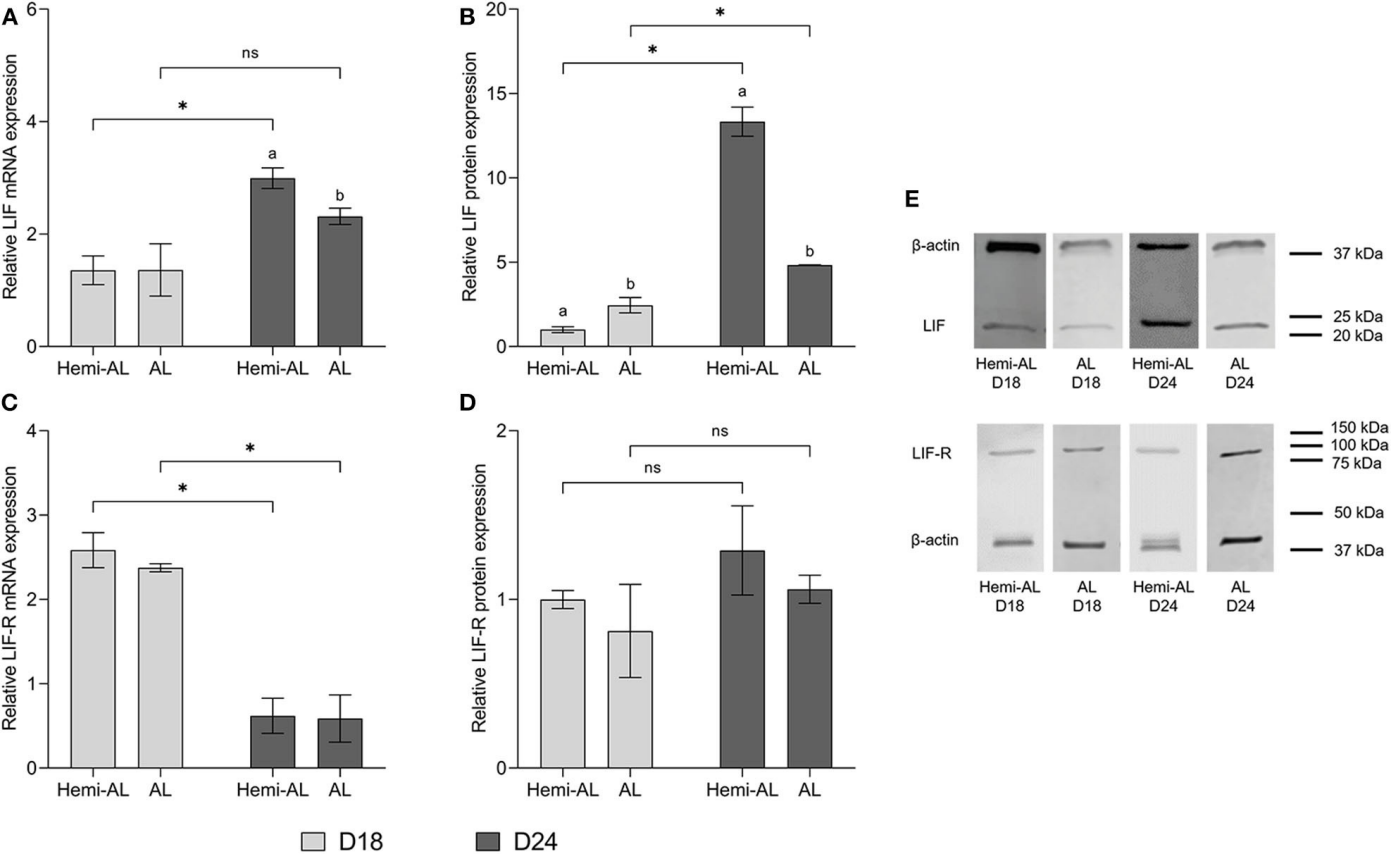
Relative mRNA **(A,C)** and protein expression **(B,D,E)** of LIF **(A,B,E)** and LIF-R **(C,D,E)** analyzed in both treatments (post-cervical artificial inseminations; Hemi-AL and embryo transfers employing donor embryos; AL), and both periods of pregnancy studied [day 18 (D18) and day 24 (D24)] at the endometrial implantation sites. Data are expressed by mean ± SEM. Columns with different superscripts (a and b) indicate significant differences (*p* < 0.05) between Hemi-AL and AL groups. Bars over the columns with * indicate significant differences (*p* < 0.05) between D18 and D24 in each experimental group. Full-length blots are presented in Supplementary Figures 1–3.

No differences in LIF or LIF-R mRNA levels were found at D18 between Hemi-AL and AL groups (Figures 2A,C) at the endometrial implantation sites. There was, however, an up-regulation of LIF protein expression in AL group compared to Hemi-AL at D18 (Figure 2B). Also, we observed a down-regulation in LIF mRNA (*p* < 0.03) and protein (*p* < 0.02) expression in AL group compared to Hemi-AL group at D24 of pregnancy (Figures 2A,B,E). No differences were found in LIF-R mRNA or protein levels between groups at any period evaluated (Figures 2C–E).

### LIF and LIF-R mRNA and Protein Expression Levels Differ During the Implantation Period at the Endometrial Implantation Sites

LIF mRNA and protein levels increased significantly (*p* < 0.003 and *p* < 0.004, respectively) at D24 over D18 in Hemi-AL group (Figures 2A,B,E). AL group did not show differences in mRNA levels between these days (Figure 2A), but protein expression was higher (*p* < 0.04) at D24 than at D18 of pregnancy (Figures 2B,E). LIF-R mRNA levels dropped significantly (*p* < 0.001) on D24 in both groups compared to D18, although this was not reflected in protein levels (Figures 2C,D).

### LIF and LIF-R mRNA and Protein Expression Levels Differ Between Implantation and Non-implantation Endometrial Areas in Allogeneic Pregnancies

**Figure 3:**
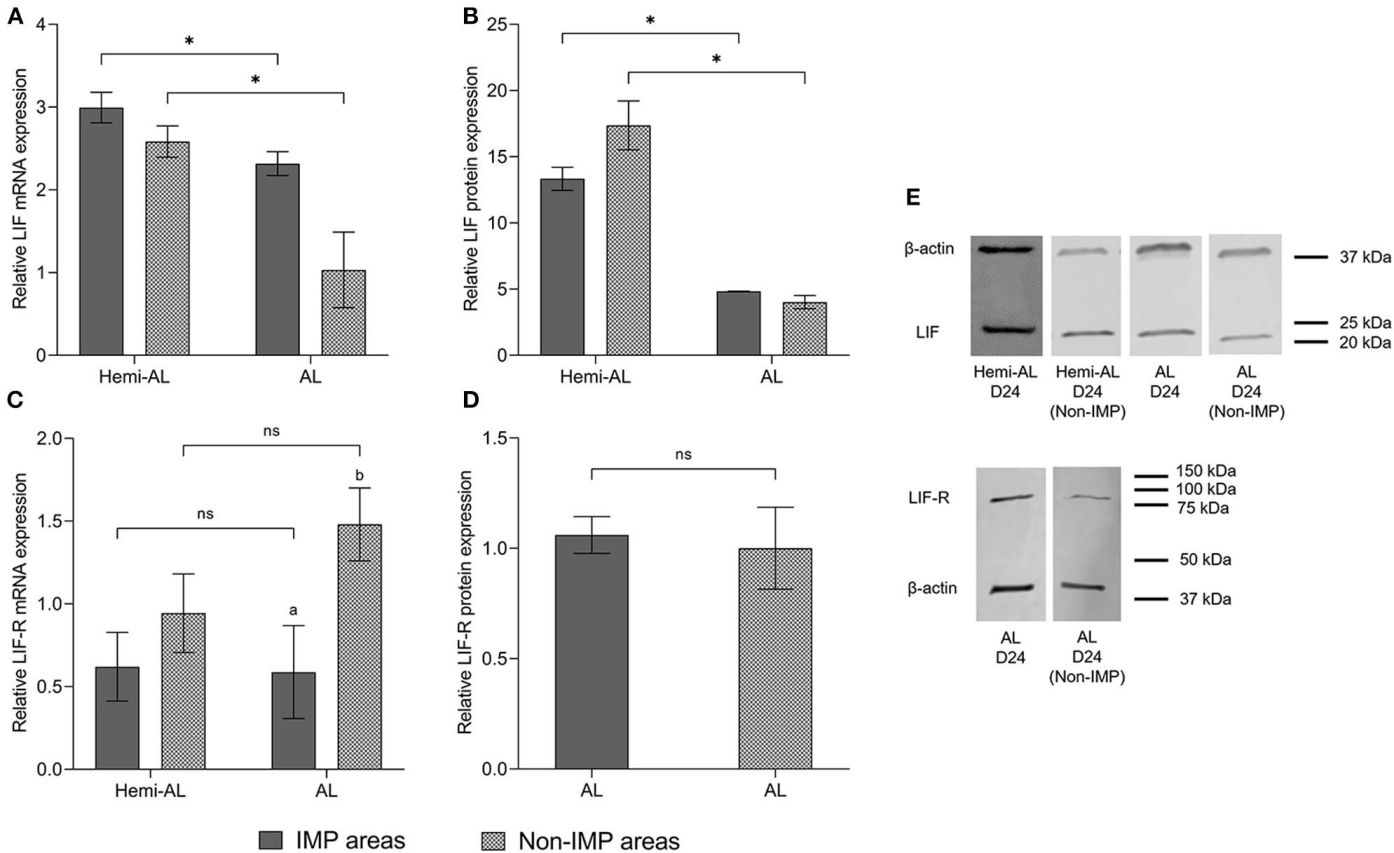
Relative mRNA **(A,C)** and protein expression **(B,D,E)** of LIF **(A,B,E)** and LIF-R **(C,D,E)** analyzed in different areas of the endometrium (Implantation areas; IMP and Non-implantation areas; Non-IMP) in both treatments (post-cervical artificial inseminations; Hemi-AL and embryo transfers employing donor embryos; AL) at day 24 (D24) of pregnancy. Data are expressed by mean ± SEM. Columns with different superscripts (a and b) indicate significant differences (*p* < 0.05) between IMP and Non-IMP areas. Bars over the columns with asterisks indicate significant differences (*p* < 0.05) between implantation Hemi-AL and AL groups within each endometrial area. Full-length blots are presented in Supplementary Figures 1–3.

Non-IMP and IMP areas showed a similar pattern for mRNA and protein expression of LIF within each group (Figures 3A,B,E). LIF-R mRNA levels showed a significant increase (*p* < 0.05) when non-implantation areas were compared to implantation areas in AL group (Figure 3C), although it was not reflected in protein levels (Figures 3D,E).

Full-length blots showing LIF and LIF-R protein levels are presented in Supplementary Figures 1, 2, respectively. Specificity of LIF and LIF-R antibody was confirmed after incubation with specific blocking peptides (Supplementary Figure 3).

### LIF and LIF-R mRNA Expression Correlate in Hemi-AL but Not in AL Endometria

**Figure 4:**
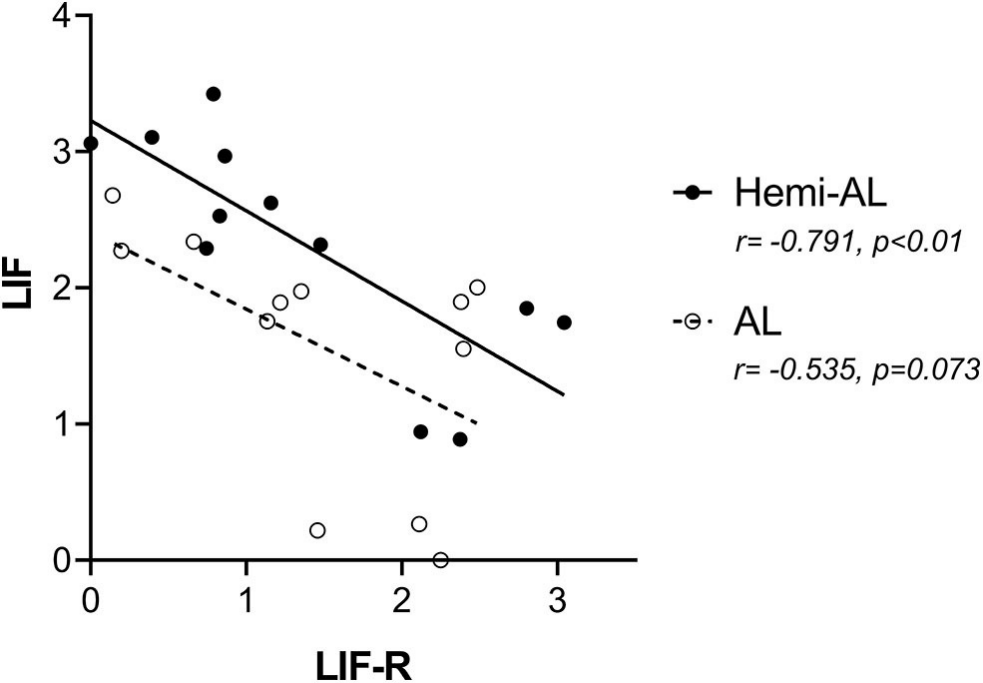
Pearson correlations of the mRNA relative expression of LIF and LIF-R between treatments (post-cervical artificial inseminations; Hemi-AL and embryo transfers employing donor embryos; AL) at both periods of pregnancy studied (days 18 and 24).

LIF and LIF-R mRNA expression showed a negative correlation in Hemi-AL group (both at D18 and D24) (*r* = −0.791; *p* < 0.01). On the contrary, in AL group LIF and LIF-R mRNA levels were not significantly correlated (*r* = −0.535; *p* = 0.073) (Figure 4).

### LIF and LIF-R mRNA Expression Do Not Correlate to the Number of Embryos Present in the Uterus

**Figure 5:**
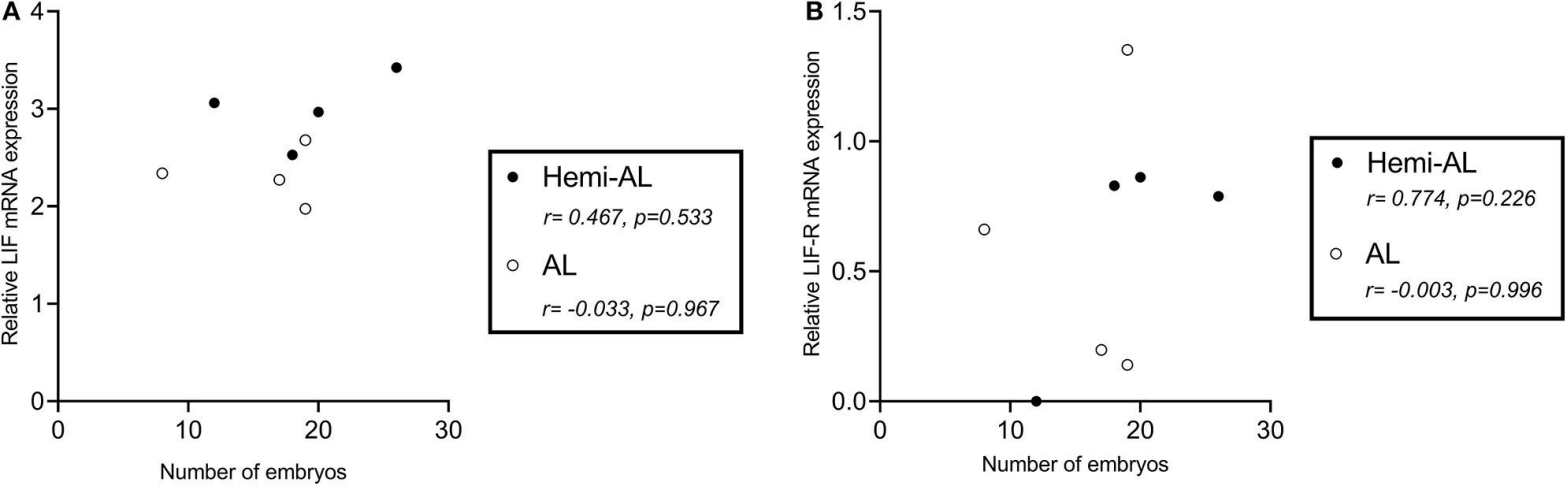
Pearson correlations of the mRNA relative expression levels of LIF **(A)** and LIF-R **(B)** at the endometrial implantation areas at day 24 in both treatments (post-cervical artificial inseminations; Hemi-AL and embryo transfers employing donor embryos; AL) and the number of collected embryos per sow at the same day.

LIF and LIF-R mRNA expression levels did not correlate significantly with the number of embryos recovered from sows after Hemi-AL (*r* = 0.467 and *p* = 0.533 for LIF; *r* = 0.774 and *p* = 0.226 for LIF-R) or AL (*r* = −0.033 and *p* = 0.967 for LIF; *r* = −0.003 and *p* = 0.996 for LIF-R) at D24 (Figure 5).

## Discussion

This study describes the endometrial expression of LIF and its receptor during the implantation period in pigs bred by ET employing donor embryos, a situation not explored before, as far as we can document. Our results, considering the use of conventional AI, confirm previous reports (13, 19–21, 28), indicating that both LIF and LIF-R are expressed in the pig endometrium during the implantation period of pregnancy.

The dynamics described for LIF expression in the available bibliography, indicate that LIF levels are physiologically low during early pregnancy starting to rise from day 12, reaching a maximum peak on day 90 (21). Our results are in general agreement with these findings in endometrium from D24 over D18. LIF-R expression in pregnant sows during the implantation period reaches maximum rates around day 15 post-insemination to eventually decrease thereafter (21). Our results agree with this report, with LIF-R expression in both hemi-allogeneic and allogeneic groups being considerably reduced on D24 compared to D18. However, this expression was not apparent in the LIF-R protein levels.

We have hereby demonstrated that LIF gene and protein expression is downregulated on D24 in ET pregnancies compared to those obtained by AI. This means that embryos (allogeneic in the case of ETs employing donor embryos and hemi-allogeneic in the case of AIs) have the potential to influence the uterine milieu at the time of implantation, and subsequently determine the fate of pregnancy. This dysregulation could be responsible for the differences in embryo survival at D24 found in a previous study conducted in our laboratory (8) where allogeneic ET sows presented higher percentages of embryo losses and delayed fetuses (54.5 and 22.2%, respectively) in comparison to hemi-allogeneic AI sows (23.4 and 5.3%, respectively) at D24 of pregnancy. These results are not surprising, given the pivotal role of LIF during pregnancy. Moreover, we found that the number of embryos recovered in each group was not significantly correlated with the LIF or LIF-R gene expression levels indicating that, it was not the number of embryos present in the uterus what altered the expression of these cytokines, but rather the allogeneic character of the embryos. These results, considering the pig as research model, could be interesting in the human species because of the current upward trend in allogeneic pregnancies due to oocyte donation and surrogacy.

In successful pregnancies, a complex modulation of the maternal immune system that avoids the rejection of the fetus and the placenta takes place, by the establishment of immunological mechanisms to develop a status of tolerance for paternal antigens (29). However, in the case of allogeneic embryos this development of immunotolerance is a major challenge because the maternal component of the embryo is also foreign to the recipients. LIF apparently acts by regulating immunotolerance during pregnancy through two strategies. On one hand, it seems to induce an anti-inflammatory response in the uterus while, on the other hand, it apparently favors embryonic mechanisms for the activation of the maternal immune response. Thus, LIF induces the differentiation of endometrium macrophages into an anti-inflammatory/regulatory phenotype (30) by inhibiting the signaling pathways STAT-1 and STAT-5 (mediated by IFNγ and GM-CSF activation) and by promoting the anti-inflammatory pathway STAT-3(31). These differentiated macrophages have a reduced expression of pro-inflammatory cytokines such as TNF-α and an impaired cytotoxic function and conversely, an increased expression of the anti-inflammatory cytokine IL-10; which has been found by us being lower in allogeneic than in hemi-allogeneic porcine pregnancies (8). LIF also appears immunomodulatory for the sub-set of NK lymphocytes (32) as shown by their increased migration into the endometrium in LIF knock-out mice (14). Therefore, one could expect that in sows with lower levels of LIF, the NK lymphocyte population might also be altered, although specific studies are needed to confirm this assumption. Finally, another subpopulation of leukocytes specially impacting on the regulation of immunotolerance is the subset of T lymphocytes known as regulatory T cells (T_reg_). These cells are programmed for depressing/suppressing the immune response against certain antigens to maintain immune homeostasis (33). T_reg_ cells secrete LIF in response to antigenic stimulation, supporting the idea that this cytokine would play an essential function in the regulation of tolerance to transplants (34). Likewise, LIF treatment increased the number of differentiated T_reg_ cells by modifying the LIF/IL-6 balance and reducing the symptoms of an autoimmune disease such as multiple sclerosis (35). In addition, LIF would lead the differentiation of naïve T cells toward the immunotolerance phenotype represented by the T_reg_ cells by supporting the expression of their specific transcription factor Foxp3 (36).

The second strategy to favor immunotolerance during pregnancy relates to embryo mechanisms. It seems that LIF could also act in certain embryo-derived factors that influence the immune system during pregnancy. In particular, LIF affects the expression of the human leukocyte antigen complex type G (HLA-G) after exposure of JEG-3 cell lines to this cytokine (37). The HLA-G is a non-classical molecule belonging to the major histocompatibility complex (MHC) class I, which is expressed by preimplantation human embryos (38) and trophoblast (39). This molecule would be implicated in the suppression of immune cell functions, such as cytolysis mediated by NK (40) and cytotoxic T lymphocytes (41), having an extensive role in the induction of immunotolerance during pregnancy. In sum, it seems that the lower levels of LIF in allogeneic pregnancies could cause a disruption of the local population of leukocytes present in the maternal-fetal interface, leading them to an immunoreactive state that is not conducive to implantation and may contribute to the high embryonic mortality rates described in transfer pregnancies.

Another interesting finding of the present study was the negative correlation observed between LIF and LIF-R gene expression. Although the regulatory mechanisms of LIF-R are quite complex (42), LIF-R is apparently downregulated in response to different cytokines and growth factors -including LIF- in different cell lines (43). This idea is consistent with the comparison made between LIF and LIF-R levels in the present study, where a significant inverse correlation between both mRNA levels is observed in hemi-allogeneic pregnancies. However, this correlation was not significant in allogeneic pregnancies, which can be interpreted as an alteration in the dynamics between the cytokine and its receptor.

In this study we also considered whether the different areas of the endometrium and whether their proximity to the embryo could exert differences in the levels of LIF or its receptor. The expression of LIF was not different between implantation and non-implantation areas in both Hemi-AL and AL groups, suggesting the secretion of LIF mostly relates to the characteristic elongation and expansion of the trophoblast, which defines the non-invasive, central “implantation” in pigs (44). Although LIF can be secreted by a wide variety of cell types, it seems that T and NK lymphocytes play an important role in activating LIF in the endometrium (45). It has been previously reported that the population of T (46), and NK lymphocytes (32) does not vary between these areas during the 20th, 30th, and 40th days of physiological pig pregnancies, which could explain why LIF expression did not differ between pig endometrial areas within the same group. However, we observed a significant downregulation of mRNA and protein expression of LIF in non-implantation areas from AL group compared to those retrieved from Hemi-AL group, reinforcing the dynamic of LIF expression observed in implantation areas between both groups. It would be interesting to investigate in future studies by immunohistochemistry the distribution patterns of LIF and its receptor in the endometrial tissue, in order to elucidate if the protein expression among the different cell types could influence the results.

## Conclusions

In conclusion, we can hypothesize that the downregulation of LIF described in allogeneic sows might lead to a status of immuno-rejection during the implantation period that hinders the advancement of normal pregnancy and contributes to the already documented embryo mortality in this species. This study was limited to a number of four animals per group to find a good balance between statistical power and the Reduction principles of the 3Rs on animal experimentation (Replacement, Reduction and Refinement). Future work should investigate whether LIF administration could be an effective therapy to improve ET in pigs.

## Data Availability Statement

The raw data supporting the conclusions of this article will be made available by the authors, without undue reservation.

## Ethics Statement

All animal procedures complied with the European Directive 2010/63/EU for animal experiments and were a priori examined and approved by the Ethical Committee for Experimentation with Animals of Murcia University (research code: 522/2019).

## Author Contributions

HR-M and CM: conceptualization. JC, IP, MG, CC, EM, AJ-M, MA-R, and CM: methodology. JC: writing—original draft preparation. AJ-M, MA-R, EM, HR-M, and CM: writing—review and editing. HR-M and CM: supervision. HR-M: project administration. MG, EM, MA-R, CM, and HR-M: funding acquisition. All authors contributed to the article and approved the submitted version.

## Conflict of Interest

The authors declare that the research was conducted in the absence of any commercial or financial relationships that could be construed as a potential conflict of interest.
