# Supplementary material for: Allogeneic Embryos Disregulate Leukemia Inhibitory Factor (LIF) and Its Receptor in the Porcine Endometrium During Implantation
Source: Front Vet Sci. 2020 Nov 24;7:611598. doi: 10.3389/fvets.2020.611598 (PMC7732548; doi:10.3389/fvets.2020.611598)
Supplement: Supplementary file 1 [file Data_Sheet_1.PDF]

Allogeneic embryos dysregulate leukemia inhibitory factor (LIF) and its receptor in the porcine endometrium during implantation

JM Cambra[1,2,3], A Jauregi-Miguel[3,4], M Alvarez-Rodriguez[3], I Parrilla[1,2], MA Gil[1,2], EA Martinez[1,2], C Cuello[1,2*], H Rodriguez-Martinez[3], CA Martinez[3].

[1]*Department of Medicine and Animal Surgery, Faculty of Veterinary Medicine, International Excellence Campus for Higher Education and Research "Campus Mare Nostrum", University of Murcia, 30100, Murcia, Spain.*
[2]*Institute for Biomedical Research of Murcia (IMIB-Arrixaca), Campus de Ciencias de la Salud, Carretera Buenavista s/n, 30120 El Palmar, Murcia, Spain.*
[3]*Department of Biomedical & Clinical Sciences (BKV), BKH/Obstetrics & Gynaecology, Faculty of Medicine and Health Sciences, Linköping University, SE-58185 Linköping, Sweden.*
[4]*Wallenberg Centre for Molecular Medicine, Linköping University*

*Correspondence: ccuello@um.es (Cristina Cuello)

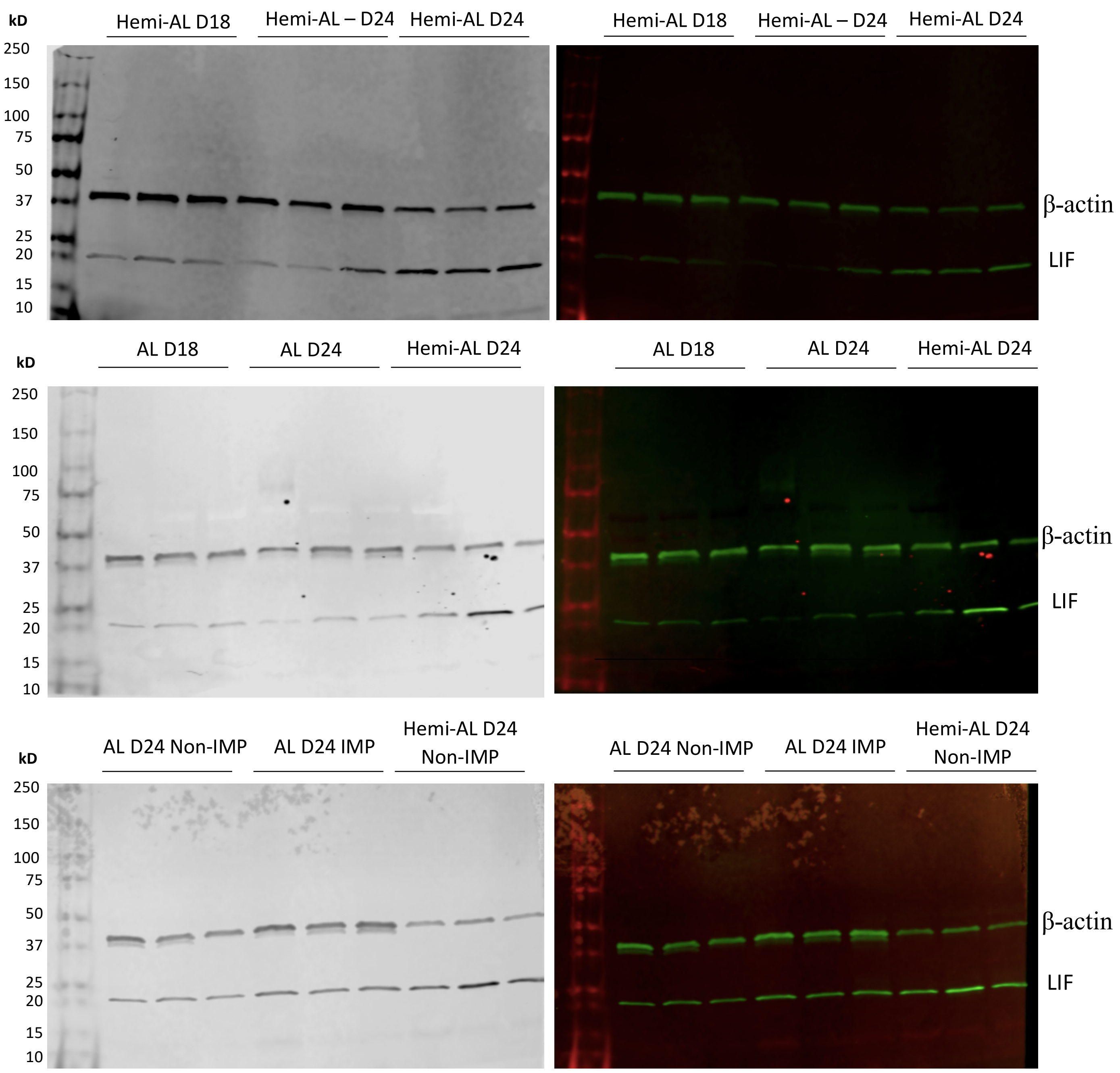

Supplementary Figure S1. Full-length blots (left side gray scale, and right side 2-color scale) showing LIF and β-actin protein expression in the different treatments (post-cervical artificial inseminations; AI/Hemi-AL and embryo transfers employing donor embryos; ET/AL), both periods of pregnancy studied [day 18 (D18) and day 24 (D24)], and different areas of the endometrium (Implantation areas; IMP and Non-implantation areas; Non-IMP).

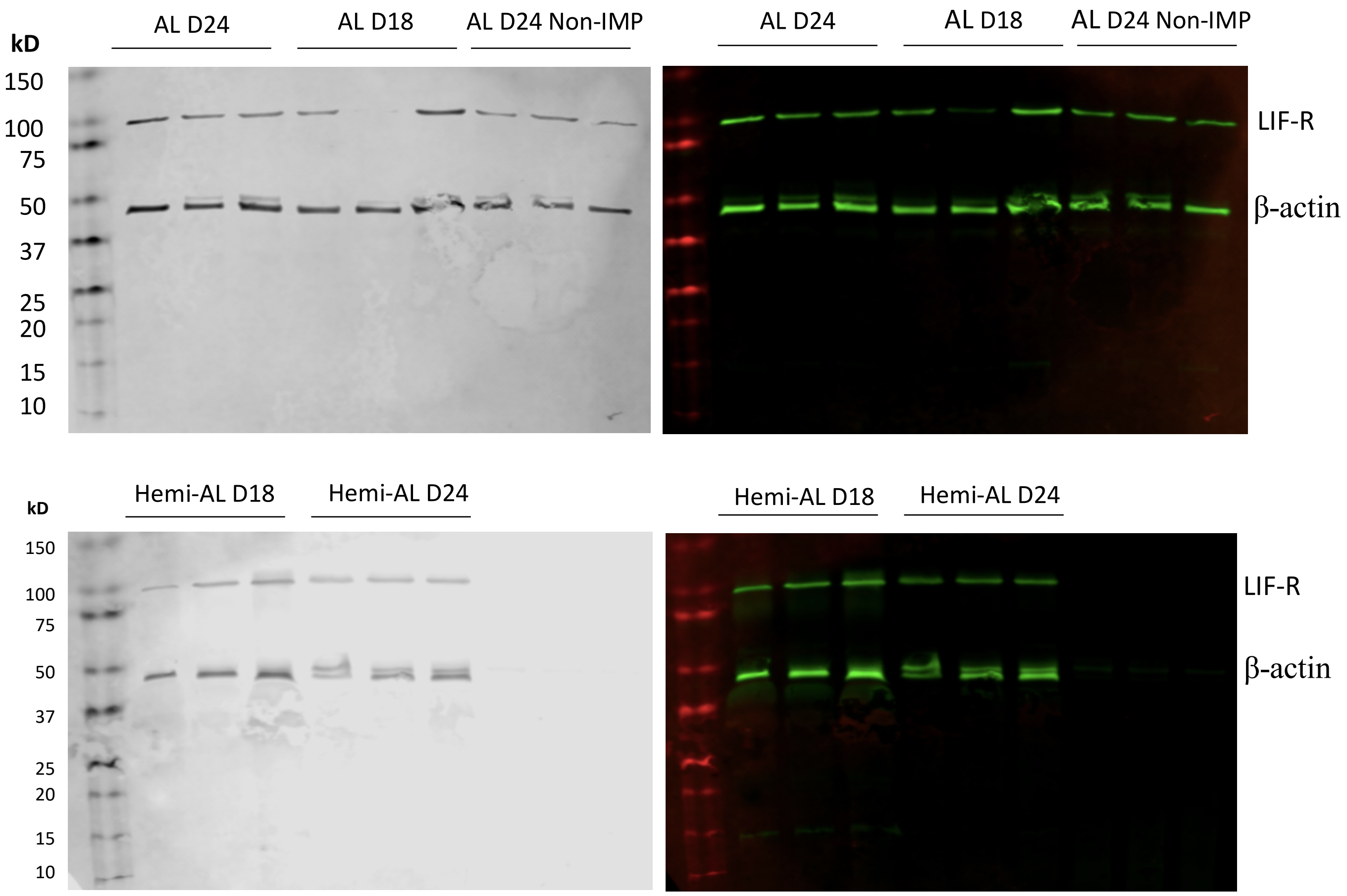

Supplementary Figure S2. Full-length blots (left side gray scale, and right side 2-color scale) showing LIF-R and β-actin protein expression in the different treatments (post-cervical artificial inseminations; AI/Hemi-AL and embryo transfers employing donor embryos; ET/AL), both periods of pregnancy studied [day 18 (D18) and day 24 (D24)], and different areas of the endometrium (Implantation areas; IMP and Non-implantation areas; Non-IMP).

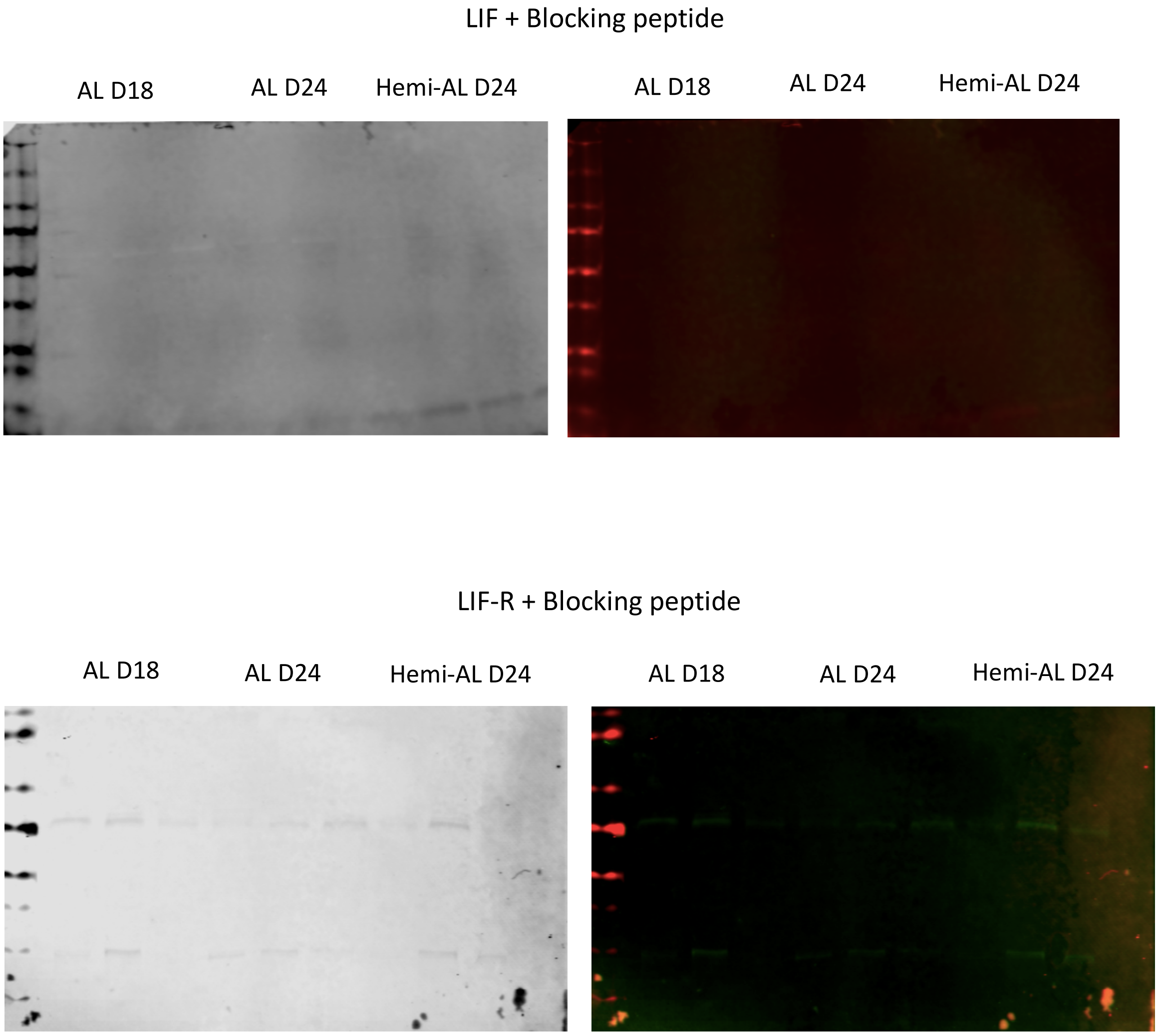

Supplementary Figure S3. Full-length blots where samples were incubated with the specific blocking peptide for LIF and LIF-R antibodies. Full-length blots (left side gray scale, and right side 2-color scale) where samples were incubated with the specific blocking peptide for LIF and LIF-R antibodies. AI/Hemi-AL: post-cervical artificial inseminations; ET/AL: embryo transfers employing donor embryos; D18: day 18 of pregnancy; D24: day 24 of pregnancy.
